# “Hunting with a knife and … fork”: Examining central coherence in autism, attention deficit/hyperactivity disorder, and typical development with a linguistic task

**DOI:** 10.1016/j.jecp.2010.06.003

**Published:** 2010-12

**Authors:** Rhonda Booth, Francesca Happé

**Affiliations:** MRC Social, Genetic, and Developmental Psychiatry Centre, Institute of Psychiatry, London SE5 8AF, UK

**Keywords:** Weak central coherence, Sentence completion, Inhibition, Autism spectrum disorders, Typical development, Attention deficit/hyperactivity disorder

## Abstract

A local processing bias, referred to as “weak central coherence,” has been postulated to underlie key aspects of autism spectrum disorder (ASD). Little research has examined whether individual differences in this cognitive style can be found in typical development, independent of intelligence, and how local processing relates to executive control. We present a brief and easy-to-administer test of coherence requiring global sentence completions. We report results from three studies assessing (a) 176 typically developing (TD) 8- to 25-year-olds, (b) individuals with ASD and matched controls, and (c) matched groups with ASD or attention deficit/hyperactivity disorder (ADHD). The results suggest that the Sentence Completion Task can reveal individual differences in cognitive style unrelated to IQ in typical development, that most (but not all) people with ASD show weak coherence on this task, and that performance is not related to inhibitory control. The Sentence Completion Task was found to be a useful test instrument, capable of tapping local processing bias in a range of populations.

## Introduction

Autism and autism spectrum disorder (ASD) are characterized not only by social and communicative deficits but also by restricted interests and activities and an uneven profile of cognitive abilities in which remarkable talents in certain visuospatial and memory tasks are notable (e.g., Jolliffe & Baron-Cohen, 1997; Shah & Frith, 1983, 1993). Current cognitive accounts of ASD have focused primarily on the areas of impairment, proposing as explanations deficits in “theory of mind,” executive functions, and so forth. Attempting to address the *assets* seen in ASD, the “weak central coherence” account proposes that people with ASD show (alongside sociocognitive deficits) a bias in cognitive style. Specifically, *coherence* refers to the tendency to integrate information in context for higher level meaning or gestalt, often at the expense of attention to or memory for featural information. People with ASD appear to show “weak” coherence, attending preferentially to details, apparently at the expense of meaning, gist, and gestalt (perhaps essential for social cognition) (Joseph, Keehn, Connolly, Wolfe, & Horowitz, 2009). Weak coherence is postulated to lie at the root of characteristic ASD symptoms such as insistence on sameness, attention to parts of objects, and uneven cognitive profile, including savant skills (see Happé & Frith, 2006). Indeed, Kanner (1943), who first named the syndrome, described as central to autism an “inability to experience wholes without full attention to the constituent parts. … A situation, a performance, a sentence is not regarded as complete if it is not made up of exactly the same elements that were present at the time the child was first confronted with it” (p. 246).

The literature pertaining to coherence in ASD has grown rapidly during the past 10 years, and an exhaustive summary is beyond the scope of this article (for a review, see Happé & Frith, 2006). Superior performance on visuospatial tasks that benefit from detail focus (e.g., Block Design, Embedded Figures) has been reported in more than a dozen studies comparing ASD and matched control groups. Detail focus has been less studied in verbal tasks, but reduced use of sentence context for disambiguation of homographs is now well replicated (e.g., Frith & Snowling, 1983; Happé, 1997; Lopéz & Leekam, 2003), as is a relative lack of benefit from meaning in memory (Tager-Flusberg, 1991), and an impressive set of studies by Jolliffe and Baron-Cohen (1999, 2000) showed poor integration of verbal material (e.g., choosing coherent bridging sentences, recognizing rare context-dependent sentence meanings) even in adults with Asperger syndrome.

The current working model of central coherence (Happé & Booth, 2008; Happé & Frith, 2006) is that a continuum of cognitive style may exist in the general population, from strong coherence (tendency to miss details and concentrate on gist) to weak coherence or detail focus (good proofreading and memory for details and verbatim information). On this conceptualization, people with ASD lie at the extreme detail-focused end of the normal continuum. One demonstration that individual differences in coherence can be found in nonclinical groups, comes from the study of the broader autism phenotype. Autism is a strongly genetic condition, and first-degree relatives who share part of the genetic loading for ASD, or some of its elements (Happé, Ronald, & Plomin, 2006), would also be likely to show a detail-focused cognitive style. Happé, Briskman, and Frith (2001) used visuospatial and verbal tests of coherence in their study of the broader autism phenotype and showed that the tendency for detail-focused processing (often leading to superior performance) was characteristic of parents (especially fathers) of boys with ASD in comparison with parents of boys with dyslexia or with typical development. However, more evidence is needed concerning the hypothesis that individual differences in coherence exist within typically developing (TD) samples.

The study of local versus global processing in typical development has a long history across diverse areas such as visual–perceptual processing (e.g., Kimchi, 1992), visuospatial construction (e.g., Akshoomoff & Stiles, 1995), music perception (e.g., Takeuchi & Hulse, 1993), and coherence and comprehension in language (e.g., Gernsbacher, 1993). In general, this research has explored children’s ability to perceive parts or wholes during different stages of development. Far less research has examined possible individual differences in children’s approach to global–local tasks. A notable exception is the study of looking time during infancy. Individual variations have been found in the amount of time infants tend to fixate on visual stimuli, which may reflect differences in visual information processing. “Short-duration” infants are suggested to take in information in a global to local sequence, whereas “long-duration” infants may process information immediately at a local level and perform a feature-by-feature analysis (e.g., Frick, Colombo, & Allen, 2000). However, fixation duration during infancy is associated with later general cognitive ability, with longer durations being associated with lower intellect (Colombo & Mitchell, 1990); thus, it is debateable whether this measure captures cognitive *style* rather than ability. The aim of our first study, then, was to attempt to measure individual differences in central coherence in a TD group and to establish whether differences in detail focus could be disentangled from differences in intellectual ability.

The specific test of central coherence used in this article paper is a simple and easy-to-administer verbal task in which participants are asked to complete sentence stems such as “You can go hunting with a knife and ….” Globally meaningful completions such as “catch a bear” show intact or strong coherence, whereas local completions such as “fork” suggest weak coherence or a tendency to prefer local over global coherence. This task has been shown to be sensitive to individual differences among parents of boys with ASD (Happé et al., 2001) and young adults with ASD (Losh et al., 2009) as well as other clinical groups with detail-focused characteristics such as women with eating disorders (Lopez, Tchanturia, Stahl, & Treasure, 2008; Lopez et al., 2008). However, data from children with typical development and from children with ASD have not been presented previously. The aim of our second study was to establish the extent to which weak coherence on this task characterizes individuals with ASD compared with an age- and IQ-matched control group.

The aim of the third study was to test an alternative explanation for detail-focused performance in terms of executive dysfunction. A number of authors (e.g., Harris & Leevers, 2000; Rinehart, Bradshaw, Moss, Brereton, & Tonge, 2000) have suggested that difficulties in global processing in ASD may reflect impairments in executive functions such as set shifting (from local to global), planning ahead (e.g., for good configuration in visuospatial tasks), and inhibitory control (e.g., over a local response). One way to test this hypothesis is to assess local–global processing in other clinical groups known to show impaired executive functions (e.g., Booth, Charlton, Hughes, & Happé, 2003). The most notable such group comprises those with attention deficit/hyperactivity disorder (ADHD), who are known to show poor planning, shifting, and monitoring as well as, in particular, marked impulsivity (e.g., Wodka et al., 2007). In the current article (Study 3), the performance of young people with ASD and those with ADHD was contrasted to test the alternative hypothesis that executive dysfunction (specifically disinhibition) results in apparent local processing bias on this test of weak central coherence.

In this article, we present for the first time the full stimuli and simple scoring criteria for the Sentence Completion Task and address three aims: (a) to test the hypothesis of individual differences in coherence style independent of general intellectual ability (Study 1 reports sentence completion data from a large TD sample spanning a wide age range), (b) to test the hypothesis of weak coherence in ASD (Study 2 reports data from well-matched samples of children with and without ASD), and (c) to test the hypothesis that local completions on our task reflect cognitive style rather than cognitive deficits in inhibitory control (Study 3 reports data from boys with ASD and boys with ADHD on the Sentence Completion Task and a standard test of impulsivity).

## Study 1: Sentence completion in typical development

Sentence completion tests have a long history, having been used as projective or personality assessment instruments (Beech & Graham, 1967; Holaday, Smith, & Sherry, 2000) and to facilitate verbal responses from severely disturbed and disabled children (Cobrinik, 1977; Sudhalter, Maranion, & Brooks, 1992). For example, in an early study by Beech and Graham (1967), a sentence completion task was found to differentiate aggressive children from nonaggressive children, whereas task performance was not significantly influenced by gender, age, or intelligence in their sample of TD children (12–15 years of age). Although completion norms for standard sentence stems have been collected, these are primarily from adult populations (e.g., Bloom & Fischler, 1980). Few studies have examined developmental effects on types of completions given.

Our aim in Study 1 was to gather normative data on our Sentence Completion Task (Happé et al., 2001), and to establish whether this test could assess individual differences in cognitive *style* rather than ability, by examining the relationship between completion performance and IQ. Second, we sought to map how completion type, and specifically local processing bias, might change with age.

Lastly, we were interested in whether gender differences on the task would be evident. There has been a suggestion that males may, in general, be more biased toward local processing than females, as shown, for example, by superior performance on the Embedded Figures Test (Witkin, Oltman, Raskin, & Karp, 1971). Baron-Cohen and colleagues (e.g., Baron-Cohen, 2002) have suggested that autism is an extreme form of the “male brain” specialized for “systemizing” over “empathizing” and have claimed that good local processing is a necessary prerequisite for superior systemizing. However, most studies of sex differences to date have tested detail focus in visuospatial tasks and have confounded superior spatial skills with superior local processing (but see Kimchi, Amishav, & Sulitzeanu-Kenan, 2009). The current verbal task, therefore, allows a somewhat fairer test of the hypothesis of sex differences in global–local processing.

### Method

#### Participants

The TD sample consisted of 176 individuals (82 males and 94 females) between 8 and 25 years of age (*M* = 14.5 years, *SD* = 4.3). School-aged participants were recruited from three secondary schools and two primary schools. Adult participants were recruited through advertisements placed in job centers, public libraries, youth clubs, hospital notice boards, and shop windows. Participants were required to have English as a first language, no clinically significant impairment or diagnosis, and no family history of ASD. Participants spanned a wide range of ethnic backgrounds and socioeconomic status (SES), but the majority were of White British origin and average SES for southern Great Britain.

All participants had a full-scale IQ (FIQ) above 70 (*M* = 107.5, *SD* = 13.4, range = 73–137) as assessed by the Wechsler Intelligence Scale for Children (WISC-III) (Wechsler, 1992) or the Wechsler Adult Intelligence Scale (WAIS-III) (Wechsler, 1997) using a short form of four subtests (Information, Picture Completion, Block Design, and Vocabulary). This short form has been reported to have high reliability (Sattler, 1992). Estimates of verbal IQ (VIQ) (*M* = 110, *SD* = 14.5) and performance IQ (PIQ) (*M* = 102.7, *SD* = 13.1) were calculated from this short form. Because the Block Design subtest is regarded as a standard measure of coherence in the ASD literature (Shah & Frith, 1993), a second FIQ score was calculated excluding the Block Design score so as to eliminate any confound with the coherence requirement of the Sentence Completion Task. None of the results was altered by substitution of this modified FIQ measure; therefore, the data presented in this article use the standard short-form FIQ score.

Participants were divided into four age groups (8–10 years, 11–13 years, 14–16 years, and 17–25 years) for comparisons (see Table 1). A chi-square test confirmed that the distribution of male and female participants across the four age groups was balanced, *χ*^2^(3, *N* = 176) = 5.87, *p* = .12, *Ф*_C_ = .18, *ns*. Age groups were comparable on all IQ measures (all *F*s < 2.13, *p* > .09). Male and female participants also did not differ by age or IQ across the whole sample (all *t*s < 1.09, *p* > .27) or within each of the four age bands (all *t*s < 1.74, *p* > .08).

#### Materials

The Sentence Completion Task consists of 14 sentence stems (see Appendix), of which 10 are designed to invite a local completion, at odds with the global coherence of the sentence, in individuals with weak central coherence. For example, a local response to the sentence “In the sea there are fish and …” would be “chips”; this response is locally coherent with the final two words in isolation but is incongruent in the context of the whole sentence. The other 4 sentence stems without this aspect of local–global conflict were used as filler items (e.g., “I was given a pen and …”).

#### Procedure

Ethical approval was obtained from the local research ethics committee of the Institute of Psychiatry at King’s College London. Informed written consent was obtained from a parent or guardian for every school-aged participant, whereas those who had left school gave their own written consent to take part. Testing for Studies 1 and 2 took place within the context of a larger study that consisted of three sessions lasting approximately 1 h. The Sentence Completion Task was administered approximately 30 min into the first session, following visuospatial and verbal tasks in both computer and pencil-and-paper formats. All participants were tested individually in a quiet room with minimal distractions.

The sentence stems were read aloud to participants by the experimenter with the instruction to say something to finish the sentence. A practice filler sentence was administered first: “He cleaned up the mess with a brush and ….” Completions produced by participants could be single words or phrases, although the experimenter did not make reference to this unless participants asked directly. The experimenter attempted to prevent participants from repeating the entire sentence in their response and, if necessary, directed them to provide only the completion. Responses were written down by the experimenter and audiotaped for later scoring. The time taken to provide a completion, measured from the end of the sentence stem to the start of the completion, was recorded to the nearest 0.5 s. The maximum time allowed for each completion was 20 s. If participants were unable to provide a completion within this time limit, a response time of 21 s was assigned. This occurred at least once for 10 participants from the 8- to 10-year group (mean occurrences in these 10 participants = 1.50, *SD* = 0.97), for 7 participants from the 11- to 13-year group (*M* = 1.29, *SD* = 0.76), for no participants from the 14- to 16-year group, and for 4 participants from the 17- to 25-year group (*M* = 1.00, *SD* = 0.00).

#### Scoring

All scoring was based on participants’ first complete response. Three dependent variables were scored for each participant: *completion score*, *number of local responses*, and *response time*.

##### Completion score

A 3-point scoring system was developed to capture the range of responses that were produced for the 10 test sentence stems: 2 points assigned for a globally meaningful completion that was produced within 10 s; 1 point assigned when the response delay was longer than 10 s, the response was an “odd” completion to the sentence but not an obviously local completion (e.g., a repetition or local associate to another word in the sentence), or when no response was provided (e.g., “don’t know”); and 0 points assigned to local responses. A local response was defined as a completion that could be expected as a response to the final two words in isolation and did *not* make sense in the context of the whole sentence. An example of a local response to the stem “The sea tastes of salt and …” would be “pepper,” whereas “water” would not be scored as a local error (even though “salt and water” might be considered as associates) because this response is appropriate to the meaning of the whole sentence. See the Appendix for scoring examples. The completion score ranged from 0 to 20.

All responses were coded by the first author. A second independent coder, who was blind to group membership, rated 33% of responses across the three studies reported in this article. Overall agreement was high (95%, kappa = .83), and disagreements between the primary and secondary coders were resolved between the two authors.

##### Number of local responses

The total number of local completions (i.e., total number of “0” responses assigned in the completion score) for each participant (maximum = 10) was used as a measure of local bias.

##### Response time

The mean response time for the 10 test stems for each participant was used as a measure of processing time (with individual response times longer than 20 s capped to 21 s) (see above).

### Results

#### Completion score

Table 2 presents results from the Sentence Completion Task for each age group in the TD sample. Age group effects were found on the completion score, with the two youngest age groups scoring significantly lower than the two oldest age groups (all *z*s > 2.08, *p* < .05,^1^ Mann–Whitney *U*). Males and females overall, and within each age group, did not differ statistically on the completion score (all *z*s < 1.29, *p* > .20); however, the effect of age group was found in males (*χ*^2 ^= 10.49, *p* = .02) but not in females (*χ*^2 ^= 4.17, *p* = .24, Kruskal–Wallis tests). Again, males in the two youngest age groups scored significantly lower than males in the two oldest age groups (all *z*s > 2.24, *p* < .03).

#### Local completions

**Fig. 1:**
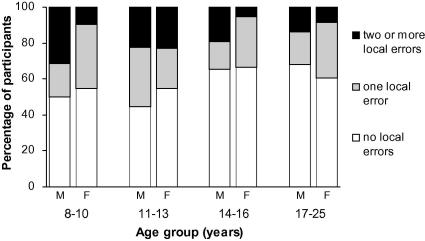
Percentage of TD male (M) and female (F) participants in each age group producing two or more, one, or no local completions on the Sentence Completion Task in Study 1.

No age group effects were found for number of local completions made. Categorical analyses comparing numbers of participants who produced one or no local errors versus two or more local errors also showed no effect of age group: all participants, *χ*^2^(3, *N* = 176) = 2.29, *p* = .52, *Ф*_C_ = .11, *ns*; males, *χ*^2^(3, *N* = 82) = 1.81, *p* = .61, *Ф*_C_ = .15, *ns*; females, *χ*^2^(3, *N* = 94) = 3.57, *p* = .31, *Ф*_C_ = .19, *ns*. Males tended to make more local completions than females (*M* = 0.82, *SD* = 1.31 vs. *M* = 0.55, *SD* = 0.76), but this difference was not statistically significant (*z* = 0.54, *p* = .59). The percentage of male and female participants in each age group who produced two or more, one, or no local errors is presented in Fig. 1. This figure provides an indication of normative performance on the Sentence Completion Task for individuals from 8 to 25 years of age.

#### Response time

The mean time for participants to provide a completion for test stems showed a significant effect of age group; the youngest group took significantly longer to provide a response than all older groups (all *z*s > 2.41, *p* < .03). The average response time for the 14- to 16-year group was also significantly quicker than the average response times for both the 11- to 13-year group (*z* = 4.00, *p* < .01) and the 17- to 25-year group (*z* = 1.99, *p* = .05). Males and females did not differ in response time to complete test stems overall or within each age group (all *z*s < 1.57, *p* > .10).

#### Correlations

No association was observed between IQ and Sentence Completion Task performance, with Spearman’s rho *r*_s_(176) values ranging from .02 to .03 between the completion score and all IQ indexes (all *p*s > .65). Furthermore, no correlations with IQ were significant within male participants, all *r*_s_(82) values < .06, *p* > .61, or female participants, all *r*_s_(94) values < .04, *p* > .70.

A significant positive correlation between age and the completion score was found, *r*_s_(176) = .25, *p* = .001. When dividing the sample by gender, this correlation was significant for male participants, *r*_s_(82) = .37, *p* = .001, but not for female participants, *r*_s_(94) = .14, *p* = .17 (difference of magnitude of correlations: *z_r_*_1–_*_r_*_2_ = 1.55, *p* = .12). Thus, younger males made fewer globally correct completions than older males.

Multiple regression analysis showed that a significant proportion (95%) of the variance in the completion score could not be explained by age, IQ, or gender and, therefore, may reflect individual differences in cognitive style.

### Discussion

Study 1 showed that within a large TD group, individual differences that were independent of differences in general cognitive ability (IQ) could be found on the Sentence Completion Task. Age effects were found in the completion score, with older children showing better completion performance overall. The only indication of gender differences on this task was the finding that the completion score increased with age in males only.

## Study 2: Sentence completion and local processing bias in ASD

Study 1 showed that the Sentence Completion Task was able to tap individual differences in typical development independent of IQ. The weak coherence account of ASD predicts that individuals with ASD will show a featural processing style, as evidenced by local completions on this task.

Sentence completion tests have not been used with ASD populations, although some older work in the projective tradition may have included children who would now be diagnosed as having autism. Cobrinik (1977), for example, used a sentence completion task to study language abilities of children (12–17 years of age) with a range of psychiatric disorders, including schizophrenia and mental retardation. An analysis of error types revealed that the most common errors could be classified as “local completions,” where responses were made in relation to the immediate cue in isolation from its general context. This occurred more often when the immediate cue contained an emotional connotation (e.g., “Now dry your eyes and *stop* … hurting him”).

In testing the reading comprehension abilities of children with autism, Frith and Snowling (1983) used the Gap Test (McLeod, 1970), which could be considered as a type of written sentence completion task. Children were asked to read a passage silently and write in missing words. The authors found that children with autism were more likely to insert a word that was semantically inappropriate to the context (although often syntactically correct) compared with children with TD or with dyslexia matched on single-word reading ability. Furthermore, when provided with a choice of words, children with autism were still more likely to select a word inappropriate to the context, indicating a failure to spontaneously access the global sentence meaning.

Study 2 tested the hypothesis that young people with ASD would show more local completions and have a lower completion score than age- and IQ-matched controls on the Sentence Completion Task.

### Method

#### Participants

The ASD group was composed of 41 males (9–21 years of age, FIQ range = 49–134) with a formal diagnosis of autism (*n* = 11) or Asperger syndrome (*n* = 30). All of the children with ASD had been diagnosed independently by a qualified clinician (psychiatrist or clinical psychologist) using DSM-IV (*Diagnostic and Statistical Manual of Mental Disorders*) criteria (American Psychiatric Association, 1994). Admission to the specialist educational placements from which the participants were recruited required a formal diagnosis of autism/Asperger syndrome. In addition, any individual for whom detailed information about source of diagnosis was lacking was excluded from analysis. Participants with a diagnosis of autism were comparable in age and PIQ to those with Asperger syndrome but scored significantly lower in FIQ and VIQ, *t*(39) > 2.19, *p* < .04. Excluding the 11 participants with an autism diagnosis did not alter the findings on the Sentence Completion Task. Of the 30 participants with Asperger syndrome, 8 had comorbid ADHD. Because the findings from the Sentence Completion Task did not change with the exclusion of these 8 participants, their data were retained.

Participants were recruited from two residential schools (one specializing in Asperger syndrome and one for children with a range of special educational needs) and parent group contacts. Current FIQ data (measured within 4 years) from the WISC-III or WAIS-R were available or collected by the experimenter for 19 participants in the ASD group. Due to time constraints, 22 participants were administered a short form to obtain FIQ, VIQ, and PIQ estimates (as described in Study 1). The use of short forms to estimate IQ in ASD populations has been validated by Minshew, Turner, and Goldstein (2005).

The control group was composed of 41 males individually matched in age (range = 9–20 years) and ability (FIQ range = 44–140) to participants in the ASD group. Of these, 5 participants with moderate learning disability (MLD, the term used for intellectual impairment in the United Kingdom) were recruited from a special educational needs school to match low-functioning participants in the ASD group. The remaining control participants were members of the TD group described in Study 1.

Participants were excluded from the control group if they had fragile X syndrome or any suggestion of an ASD. As a screening measure, parents of MLD children completed the Social Communication Questionnaire (SCQ) (Berument, Rutter, Lord, Pickles, & Bailey, 1999), a brief checklist derived from the Autism Diagnostic Interview (ADI-R) algorithm items (Lord, Rutter, & Le Couteur, 1994). Participants were excluded if their SCQ score fell in the ASD range.

Participant characteristics for the ASD and control groups are presented in Table 3. Statistical comparisons confirmed that the ASD and control groups did not differ significantly in age or IQ.

#### Procedure

Test procedures and measures were identical to those in Study 1. When participants were not able to produce a completion within 20 s, the maximum of 21 s was assigned. This occurred at least once for 10 participants in the ASD group (mean occurrence for these participants = 1.30, *SD* = 0.68) and for 7 participants in the control group (*M* = 1.43, *SD* = 1.13).

### Results

#### Completion score

Table 3 presents group means for the performance measures on the Sentence Completion Task for participants in Study 2. A two-tailed Mann–Whitney *U* test found that the ASD group had a significantly lower completion score than the control group.

#### Local completions

**Fig. 2:**
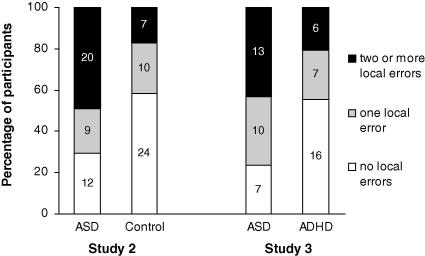
Percentage (and *n*) of participants in each age group producing two or more, one, or no local completions on the Sentence Completion Task in Studies 2 and 3.

As shown in Table 3, the ASD group produced significantly more local completions than the control group. The percentage of participants in each group who produced two or more, one, or no local responses is presented in Fig. 2. Significantly more participants in the ASD group than in the control group made two or more local completions, *χ*^2^(1, *N* = 82) = 9.33, *p* < .01, *Ф* = .34.

#### Response time

The ASD and control groups did not differ in mean time to complete test stems (see Table 3).

#### Correlations

In the control group (as in the TD sample in Study 1), there was no relation between the completion score and FIQ, *r*_s_(41) values = .04, *p* = .81. In contrast, a significant positive correlation was found in the ASD group, *r*_s_(41) = .31, *p* < .05 (no significant difference in magnitude of correlations, *z_r_*_1–_*_r_*_2_ = 1.22, *p* = .22). There were no significant correlations between the completion score and VIQ and PIQ in either group, all *r*_s_(41) values < .26, *p* > .11.

The correlation between age and the completion score was not significant in the control group, *r*_s_(41) = .25, *p* = .11, although it was of similar magnitude to that in the larger TD sample in Study 1. No association was found in the ASD group, *r*_s_(41) = −.02, *p* = .90, although the coefficients for the ASD and control groups did not differ significantly in magnitude (*z_r_*_1–_*_r_*_2_ = 1.21, *p* = .23).

### Discussion

As predicted, participants with ASD made significantly more local completions to the test stems and had a lower completion score than participants in the age- and IQ-matched comparison group. This was the case despite the low absolute number of local completions. Two factors may have contributed to the scarcity of local completions. First, our scoring system was conservative in relation to our hypothesis; that is, we did not score as “local” any completion that could possibly make sense in the whole sentence context. Thus, a completion such as “The shoemaker mended the shoes and … laces” was not scored as local because it makes sense in the context of the whole sentence even though it likely reflects attention to the final words of the stem and a relatively local processing style. Second, our ASD sample was generally high-functioning (with the majority of participants having an Asperger syndrome diagnosis) and presumably was able to recognize, to some degree, the (entirely implicit) requirement for global sense. The significant correlation between IQ and the completion score in the ASD group, but not in the control group, perhaps reflects the use of compensation strategies. Future work with low-functioning participants with ASD, or under speeded conditions, might clarify whether local completions are more common when compensatory strategies are removed.

## Study 3: Sentence completion and inhibition—ADHD and ASD group comparisons

Study 2 showed that the majority of individuals with ASD showed a local processing bias in the Sentence Completion Task compared with their age- and IQ-matched controls. Our interpretation of this predicted finding is that local completions reflect weak coherence in ASD. However, an alternative explanation is that people with ASD are less able to inhibit an automatic or prepotent response to the final part of the sentence stem and also differ from controls not in local bias but rather in poor impulse control.

Sentence completion has also been used as an executive function task, notably in the Hayling Sentence Completion Test (Burgess & Shallice, 1996), where the emphasis is on the contrast between straightforward meaningful completion (thought to be effortless) and deliberately meaningless completion (effortful inhibition) conditions. The ability to inhibit a meaningful response to sentence completions has been found to increase with development (Lorsbach & Reimer, 1997) and requires intact functions of the frontal lobes (Crawford & Henry, 2005).

It has been suggested, more generally, that some findings attributed to weak coherence might be better explained by executive dysfunction (e.g., Rinehart et al., 2000). Elsewhere (Booth et al., 2003), we have addressed, for example, the executive dysfunction explanation for fragmented drawing style (Harris & Leevers, 2000) by showing that poor planning is not sufficient to cause detail-focused drawing and that measures of local bias and of planning are not correlated in a drawing task. However, Pellicano, Maybery, and Durkin (2005) did find a correlation between tasks selected to tap central coherence and executive skills in young TD children.

It is worth noting that an impulsivity, or poor inhibitory control, account of Study 2’s findings would need to postulate that local completions are the default response, which older children and non-ASD participants manage to inhibit so as to give globally meaningful completions. By contrast, the weak coherence account suggests that local completions come to mind automatically *only* for participants with a more local processing bias (e.g., those with ASD) and that no effort of inhibition is necessary for participants with a more global processing style, for whom coherent and meaningful completions are the default.

To test the impulsivity account of poor Sentence Completion Task performance, Study 3 contrasts two clinical groups: ASD and ADHD. Individuals with ADHD are well documented to show poor inhibitory control and an impulsive response style (DSM-IV) (American Psychiatric Association, 1994; Barkley, 1997). The dysexecutive account of Study 2’s findings, therefore, would predict a high rate of local completions in an ADHD group. In addition, in Study 3 we report the relationship between performance on the Sentence Completion Task and that on a standard test of inhibitory control. Our prediction from the weak coherence account was that, despite poor impulse control, the ADHD group would make fewer local completions than the ASD group and that there would be little relation between Sentence Completion Task errors and errors of commission on the inhibition task.

### Method

#### Participants

The ASD group was composed of 30 boys with a formal diagnosis of either high-functioning autism (*n* = 6) or Asperger syndrome (*n* = 24) recruited through specialist units and parent group contacts. All of the children with ASD had been diagnosed independently by a qualified clinician (psychiatrist or clinical psychologist) using DSM-IV criteria (American Psychiatric Association, 1994). Admission to the specialist educational placements from which the participants were recruited required a formal diagnosis of autism/Asperger syndrome. In addition, any individual for whom detailed information about source of diagnosis was lacking was excluded from analysis. Furthermore, participants were excluded if they had comorbid ADHD, attention deficit disorder (ADD), hyperkinetic disorder, and/or Tourette syndrome. To demonstrate reliability of findings on the Sentence Completion Task, there was no overlap of ASD participants in Studies 2 and 3.

The ADHD group was composed 29 boys with a formal diagnosis of either ADHD (DSM-IV, *n* = 19) or hyperkinetic disorder (ICD-10 [*International Classification of Diseases*], *n* = 10) recruited through specialist referral centers. Diagnosis of ADHD was also confirmed by parent interview (Parental Account of Childhood Symptoms) (Taylor, Schacher, Thorley, & Weiselberg, 1986), and ASD participants were excluded if they showed marked hyperactivity traits on this measure (for details, see Happé, Booth, Charlton, & Hughes, 2006). Children were excluded if they had additional disorders such as pervasive developmental disorder, Tourette syndrome, and obsessive compulsive disorder. Furthermore, children with a diagnosis of ADD without the hyperactivity component were not included given suggestions that inhibitory deficits might not be characteristic of this subgroup (e.g., O’Driscoll et al., 2005). The majority of boys (*n* = 26) had been prescribed medication for the management of their ADHD. All were required to be off medication for at least 24 h prior to the administration of the experimental tasks. One exception occurred where a boy could be taken off medication only 17 h prior to assessment due to family constraints. Data from this child were included after analysis of group data excluding this participant showed no change in the pattern or significance of results. Following clinical advice, IQ assessments were conducted with children on medication because this is considered to result in a more accurate assessment of intellectual level (E. Taylor, personal communication).

Across all groups, no child was excluded on the basis of reading (5 ADHD, 1 ASD), conduct (5 ADHD), or anxiety disorder (1 ADHD, 2 ASD). Excluding participants with comorbid disorders did not change the pattern or significance of the results. All participants were between 8 and 16 years of age and had a minimum FIQ of 69 or above as assessed by the full administration of the WISC-III (Wechsler, 1992). Participant characteristics for each group are presented in Table 4. Statistical comparisons showed that groups did not differ in age or IQ.

This sample was described previously in a study of planning drawing (Booth et al., 2003) and executive function profiles across ages (Happé et al., 2006).

#### Procedure

Testing took place within the context of a larger study that consisted of two sessions of computerized and paper-and-pencil tasks lasting approximately 2 h. The Sentence Completion Task was administered during the first half-hour of the first session (using the procedure described in Study 1), whereas the Go No-Go Task was administered during the last half-hour of the second session. On the Sentence Completion Task, response times longer than 20 s (capped to 21 s) (see above) occurred at least once for 6 participants from the ADHD group (mean occurrence for these participants = 1.00, *SD* = 0.00 and for 5 participants from the ASD group (*M* = 1.40, *SD* = 0.89).

#### Go No-Go task

The Go No-Go Task from the Maudsley Attention and Response Suppression (MARS) task battery (Rubia et al., 2001) was used to measure response inhibition. The task was run through a Datalux Databrick microcomputer with a high-resolution color 10-inch touch-sensitive screen with an additional single external response button. Participants were presented with a series of aeroplanes and bombs appearing on the screen. They were told to respond as fast as possible when an aeroplane appeared but to withhold response when a bomb appeared. The task was administered in two blocks of 90 trials, of which 30% were bomb (inhibition) trials. The interstimulus interval (ISI) was 1600 ms; stimulus duration was 200 ms, followed by a blank screen of 1400 ms. The measures taken from this task were total errors of commission or “false alarms” (as a percentage of bomb trials) and total errors of omission (as a percentage of plane trials). In addition, signal detection theory was used to provide a measure of participants’ sensitivity to the task, *A’* (Grier, 1971). Computer errors that occurred at the time of testing resulted in the exclusion of several participants from the Go No-Go Task (3 ADHD, 6 ASD). Groups remained matched in age and IQ after the exclusion of these 9 participants, all *t*s(48) < 1.33, *p* > .19. Data from this task for most of the current participant groups were previously presented in the context of executive functioning composites derived from a battery of eight tasks (Happé et al., 2006). The current article presents new data describing the relationship between the Sentence Completion Task and Go No-Go Task performance.

### Results

#### Completion score

Table 4 presents group results from the Sentence Completion Task. The ASD group had a lower completion score than the ADHD group, although this difference was not significant on a two-tailed nonparametric test (*p* = .07). We retain nonparametric analyses here for consistency, but under (two-tailed) parametric analyses this group difference reached significance, *t*(57) = 2.07, *p* = .04.

#### Local completions

A two-tailed Mann–Whitney *U* test found that the ASD group produced significantly more local completions than the ADHD group. The percentage of participants in each group who produced two or more, one, or no local responses is presented in Fig. 2. A 2 × 3 chi-square analysis found this distribution of participants to be significantly different between groups, *χ*^2^(2, *N* = 59) = 6.62, *p* = .04, *Ф*_C_ = .33. More participants in the ASD group than in the ADHD group made two or more local completions, although this did not reach significance, *χ*^2^(1, *N* = 59) = 3.46, *p* = .06, *Ф* = .24, *ns*.

#### Response time

No group differences were found in the time to complete the test stems (see Table 4).

#### Correlations

No significant correlations were found between any IQ measure and the completion score in the ADHD group, all *r*_s_(29) values < .33, *p* > .08, or in the ASD group, all *r*_s_(30) values < .16, *p* > .40.

A positive correlation was found between age and the completion score that reached significance in the ADHD group, *r*_s_(29) = .39, *p* = .04, but not in the ASD group, *r*_s_(30) = .32, *p* = .08. A nonsignificant, but moderate, correlation was found between age and response time in the ASD group (with older participants taking longer to respond), *r*_s_(30) = .36, *p* = .06, but not in the ADHD group, *r*_s_(29) = .10, *p* = .60, *z_r_*_1–_*_r_*_2_ = 0.98, *p* = .33.

#### Go No-Go Task

The ADHD group showed difficulty in withholding a response to the “no-go” stimulus (bombs) and made significantly more commission errors than the ASD group (ADHD: *M* = 48.8, *SD* = 13.4; ASD: *M* = 38.8, *SD* = 20.6), *t*(39.1) = 2.02, *p* < .05. The ADHD group also made more omission errors, failing to respond to the “go” stimulus (aeroplane) more often than the ASD group, although this difference was not significant (*M* = 21.0, *SD* = 11.0 vs. *M* = 15.5, *SD* = 14.2), *t*(48) = 1.53, *p* = .13. Overall, the ADHD group was somewhat less sensitive in distinguishing the two types of stimuli as measured by *A’*, although again this difference did not reach significance (*M* = 0.73, *SD* = 0.12 vs. *M* = 0.79, *SD* = 0.15), *t*(48) = 1.77, *p* = .08.

#### Relationship between impulsivity and local completion performance

The main measure of impulsivity, errors of commission, did not correlate significantly with the completion score for either group: ASD, *r_s_*(24) = −.20, *p* = .35; ADHD, *r_s_*(26) = −.30, *p* = .14. Errors of commission also did not correlate significantly with time to provide a completion: ASD, *r_s_*(24) = −.12, *p* = .57; ADHD, *r_s_*(26) = −.24, *p* = .24.

The measure of sensitivity in discriminating between “go” and “no-go” trials (*A’*) did not correlate significantly with the completion score: ASD, *r_s_*(24) = .31, *p* = .14; ADHD, *r_s_*(26) = .35, *p* = .08. The correlation coefficients were of medium strength (Cohen, 1988); however, removing the effects of age and FIQ lessened the strength of these correlations, particularly in the ADHD group: ASD, *Pr*_age+IQ_(20) = .28, *p* = .21; ADHD, *Pr*_age+IQ_(22) = .08, *p* = .70. No significant correlation was detected between Sentence Completion Task response time and Go No-Go Task *A’* in the ASD group, *r_s_*(24) = .01, *p* = .97, or in the ADHD group, *r_s_*(26) = .31, *p* = .12, *z_r_*_1–_*_r_*_2_ = 1.20, *p* = .23.

### Discussion

As predicted, individuals in the ADHD group did not show weak coherence on the Sentence Completion Task. Despite their inhibitory difficulties, as seen in errors of commission in the Go No-Go Task, they made no more local completions than the comparable TD group (11–13 years of age) in Study 1. In addition, although individuals in the ADHD group were more impulsive than the ASD group on the executive task, they made significantly fewer local completions than the ASD participants. Thus, problems of inhibition do not appear, in themselves, to result in detail-focused performance on the Sentence Completion Task.

## General discussion

The Sentence Completion Task appears to be a useful measure of central coherence. It proved to be sensitive to individual differences independent of IQ among TD young people and capable of tapping weak central coherence in ASD groups versus ability-matched and ADHD comparison groups.

The tendency to provide local completions appears to be independent of intellectual ability, suggesting that the Sentence Completion Task taps individual differences in cognitive *style* rather than merely ability. First, among a large sample of TD participants, there was no significant relation between IQ and Sentence Completion Task performance. Second, looking across studies suggests that the control group in Study 2, which included participants with intellectual impairment (FIQs of 47–134), performed very similarly to their TD peers (14–16 years of age) in Study 1. In Study 3 as well, the ADHD group included some boys with low IQ (FIQs of 69–138), yet here too performance was very similar to that of their Study 1 TD peers (11–13 years of age). Third, in neither the control group (Study 2) nor the ADHD group (Study 3) did the number of local completions correlate significantly with IQ. The possibility of measuring detail-focused cognitive style, rather than ability, in nonclinical groups is exciting because it would allow examination of putative relationships with, for example, language learning style (holistic vs. analytic) (e.g., Richards, 1990) during the preschool years and looking time during infancy (see above).

The predicted tendency for detail-focused processing, weak coherence, was found in the ASD group. As discussed above, group differences were significant despite the fact that the actual number of local completions was relatively low (due in part, perhaps, to conservative scoring). Examination of the data suggests, however, that approximately a quarter of the ASD group did not make any local completions. This may reflect heterogeneity within ASD; in nearly all studies of cognitive characteristics in ASD, a proportion of participants fail to show the expected pattern (e.g., theory of mind performance; Happé, 1995). It is also notable that weak coherence appears to be a bias rather than an inability to process globally; thus, ASD detail focus is most evident on open-ended tasks and can be reversed where explicit instructions for global processing are given (e.g., Snowling & Frith, 1986; see discussion in Happé & Frith, 2006). Our efforts to make the Sentence Completion Task entirely open-ended with no implied “correct” answer type might not have been fully successful, at least for the higher functioning individuals with ASD. Our data provide some possible hints that, in the ASD group only, compensation strategies were needed to avoid local completions; in Study 2 the correlation between the completion score and IQ was significant in the ASD group but not in the control group, although in Study 3 this was not replicated, possibly due to the narrower IQ range.

Alternative accounts of detail focus in ASD exist. Study 3 was designed to test the dysexecutive explanation of local bias in ASD. Results showed that problems of impulse control could not account for the tendency to make local completions. The current task was not designed, however, to test between competing conceptualizations of detail focus. Indeed, our findings fit all three of the competing models currently presented in the literature. Mottron, Dawson, Soulieres, Hubert, and Burack’s (2006) “enhanced perceptual functioning” account posits overdeveloped low-level perception and atypical relationships between low- and high-level processing. Baron-Cohen’s “hyper-systemizing” account (e.g., Baron-Cohen, 2005) suggests that the ability to isolate parts is a first step in the ability to figure out the rules of closed systems—enhanced in ASD. Plaisted (2001) hypothesized that people with autism process features held in common between objects relatively poorly and process features unique to an object, that is, those that discriminate items relatively well. Each of these alternative conceptualizations favors local superiority over global weakness, in contrast to Frith’s (1989, 2003) original description of coherence. However, local completions in our task may reflect either enhanced attention to local features (final words) or reduced tendency to integrate all elements of the sentence. Further tests are needed to isolate the local and global contributions to detail focus in ASD (see Happé & Booth, 2008).

The current article has presented novel data regarding individual differences in weak coherence, their independence from IQ, and at least some aspects of executive function. However, as with all research, some limitations pertain. Regarding the Sentence Completion Task, the use of fixed order of sentence stems meant that it was impossible to separately analyze specific item effects and the effect of immediately preceding filler stems. Inspection of the data suggested that local completions were spread across all test stems and that the removal of individual sentences (e.g., the ambiguous “night/knight” stem) did not change the pattern or significance of results. However, future research should employ a counterbalanced order of stems to examine item effects and to establish whether filler stems (for which local completions were also globally congruent) encouraged local completions on proceeding test stems. In addition, a longer task with more than 10 test stems might lead to better psychometric properties; on the current (very short and simple) test, local completions were rare and the range of responses was relatively narrow. The current studies reported data from 71 individuals with ASD, but subgroup sizes were still too small to compare performance by those with autism versus Asperger syndrome. Lastly, although the TD group in Study 1 was large for a cognitive study, it remains possible that some effects, particularly sex differences, might have reached significance if larger numbers had been included.

In conclusion, the Sentence Completion Task appears to be a simple and easy-to-administer test capable of tapping local processing bias, or weak coherence, in a range of populations. Data from Study 1 present an approximate range of normative performance that may provide a useful guide for clinical use of this task within a wider assessment of strengths and difficulties in individuals with ASD. Future research examining sources of heterogeneity in cognitive style within the autism spectrum (e.g., symptom or IQ subgroups), and effects of compensation and intervention, would be worthwhile.

## Figures and Tables

**Table:** Sentence Completion Task stimuli and scoring examples.

	Sentence stems (in order of administration)	Examples of 0-point local completions	Examples of 2-point correct completions and 1-point completions^a^
1^b^	I was given a pen and …		
2	The sea tastes of salt and …	pepper/vinegar/sugar/sour	water/seaweed/sand/was cold
3	Hens lay eggs and …	bacon/chips/milk/noodles	chicks/have feathers/(eggs)
4^b^	The woman took the cup and …		
5	You can get burnt by the sun and …	moon/sea/daughter/son/sand/stars/rain	fire/hot water/it hurts
6^b^	You can feed a child bread and …		
7	Little boys grow up to be men and …	women/lady	girls grow up to be women/adults/granddads
8	In the sea there are fish and …	chips	sharks/whales/lots of sea life
9	In a cave lived a bat and …	ball	bear/spiders/a caveman
10	You can go hunting with a knife and …	fork	gun/bow and arrow
11	The old shoemaker mended the shoes and …	socks/clothes/hats/shirt	boots/soles/gave them back/cleaned them/(laces)/(slippers)
12	The fireman carried the bucket and …	spade	hose/water/ladder/put out the fire
13^b^	A vet cares for cats and …		
14	The night was black and …^c^	white/blue	dark/cold
	[The knight was black and …]		[silver/had a large sword]


a Examples of 1-point completions are in parentheses.

b Filler stem.

c Pilot work revealed that the item “The night was black and …” was sometimes construed as “The *knight* was black and …” Participants who heard the stem as referring to a “knight” might give legitimate global answers (e.g., “white”) that would have been scored as local completions for the intended “night” stem. Because of the ambiguity of this item, the experimenter asked any participant who gave such an answer whether he or she had been thinking of a “knight on a horse or a starry night” immediately after this item. For participants who answered that they had thought of a knight on a horse, answers such as “white” and “blue” were rated as global, rather than local, completions. Importantly, removing this sentence stem from analyses did not change the pattern of significant findings in any of the three studies reported in this article.

**Table 1:** Study 1: TD participant characteristics by age group and gender.

	8–10 years (*n* = 47)		11–13 years (*n* = 40)		14–16 years (*n* = 44)		17–25 years (*n* = 45)		Males (*n* = 82)		Females (*n* = 94)	
Age	9.7	(0.6)	12.3	(1.0)	15.6	(0.8)	20.4	(2.5)	14.8	(4.0)	14.2	(4.6)
FIQ	108.1	(12.4)	105.1	(15.8)	106.3	(11.9)	110.3	(13.4)	108.7	(15.0)	106.5	(11.9)
VIQ	111.6	(14.9)	106.5	(16.7)	110.4	(13.8)	111.1	(12.7)	111.2	(16.4)	108.9	(12.7)
PIQ	101.9	(11.6)	101.8	(13.7)	100.3	(10.9)	106.8	(15.3)	103.5	(13.3)	102.0	(12.9)

*Note.* Values are means and standard deviations (in parentheses).

**Table 2:** Study 1: Sentence Completion Task TD participant results by age group.

	8–10 years		11–13 years		14–16 years		17–25 years		Kruskal–Wallis *χ*^2^	*p*	Post hoc Mann–Whitney *U* test, *p* <.05
Completion score (maximum = 20)	17.47 (2.28)		17.38 (2.56)		18.52 (2.18)		18.51 (1.63)		13.26	.004	8–10, 11–13 > 14–16, 17–25
Range	8–20		10–20		10–20		13–20				

Number local completions (maximum = 10)	0.74	(1.11)	0.90	(1.28)	0.59	(1.06)	0.44	(0.69)	3.70	.30	
Range	0–6		0–5		0–5		0–2				

Response time to test stems (s)	3.91	(1.87)	3.12	(1.72)	1.89	(0.74)	2.56	(1.60)	43.43	<.0005	8–10 > 11–13, 14–16, 17–25; 11–13, 17–25 > 14–16
Range	1.25–11.60		1.05–10.10		0.80–3.90		0.70–9.75				

*Note.* Values are means and standard deviations (in parentheses).

**Table 3:** Study 2: Participant characteristics and Sentence Completion Task results by group.

	ASD (*n* = 41)		Control (*n* = 41)		*t*	*p*	Cohen’s *d*
Age	14.4	(2.6)	14.5	(2.7)	0.21	.84	.05
FIQ	95.0	(21.6)	95.6	(22.1)	0.11	.92	.02
VIQ	97.3	(21.3)	97.8	(21.9)	0.11	.92	.02
PIQ	94.3	(20.4)	94.6	(19.1)	0.08	.93	.02
Completion score (maximum = 20)	15.63	(2.80)	17.41	(2.80)	3.10^a^	.002	.69
Range	8–20		10–20				
Number local completions (maximum = 10)	1.56	(1.38)	0.76	(1.20)	3.00^a^	.003	.67
Range	0–5		0–5				
Response time to test stems (s)	3.54	(2.17)	3.33	(2.35)	0.76^a^	.45	.17
Range	0.70–10.10		0.70–11.60				

*Note.* Values are means and standard deviations (in parentheses).

a Mann–Whitney *U* tests, *z* values reported.

**Table 4:** Study 3: Participant characteristics and Sentence Completion Task results by group.

	ASD (*n* = 30)		ADHD^a^ (*n* = 29)		*t*	*p*	Cohen’s *d*
Age	11.0	(2.5)	11.7	(1.7)	1.34	.19	.36
FIQ	97.3	(16.7)	98.9	(17.9)	0.35	.73	.09
VIQ	100.3	(16.5)	99.1	(18.7)	0.27	.79	.07
PIQ	94.4	(16.2)	97.9	(15.0)	0.85	.40	.23
Completion score (maximum = 20)	14.98	(3.79)	16.79	(2.77)	1.82^b^	.07	.55
Range	7–20		11–20				
Number local completions (maximum = 10)	1.77	(1.72)	0.90	(1.29)	2.39^b^	.02	.58
Range	0–6		0–4				
Response time to test stems (s)	3.79	(2.03)	3.36	(1.33)	0.53^b^	.60	.25
Range	1.15–10.90		1.25–6.20				

*Note.* Values are means and standard deviations (in parentheses).

a The ADHD group did not differ from a subgroup of male controls selected from Studies 1 and 2 to match for age and FIQ on the three indexes from the Sentence Completion Task (all *p*s > .14).

b Mann–Whitney *U* tests, *z* values reported.

## Footnotes

1 All reported *p* values are two-tailed.
